# NAFLD and Hypothyroidism: Deciphering Pivotal Genetic Variants, Cellular Expression Landscapes, and Spatial Architectures

**DOI:** 10.3390/ijms27041850

**Published:** 2026-02-14

**Authors:** Ruiqi Zhao, Mengyao Han, Minling Lv, Sen Lin, Ximing Wang, Jing Li, Jialing Sun, Xiaozhou Zhou

**Affiliations:** 1 The Fourth Clinical Medical College, Guangzhou University of Chinese Medicine, Shenzhen 518033, China; zhaoruiqi1998@163.com (R.Z.); mulinsen031@163.com (S.L.); wxmdyx0921@163.com (X.W.); lilijingping@163.com (J.L.); suangenlingzi1992@126.com (J.S.); 2 The Second Clinical Medical College, Guangzhou University of Chinese Medicine, Guangzhou 510120, China; 17719996436@163.com; 3 Faculty of Chinese Medicine, Macau University of Science and Technology, Taipa, Macao 999078, China; luxlyulyu@163.com

**Keywords:** NAFLD, hypothyroidism, single-cell transcriptomics, spatial transcriptomics, GWAS, chromatin accessibility

## Abstract

Thyroid hormones profoundly modulate hepatic fatty acid and cholesterol synthesis and turnover. Although nonalcoholic fatty liver disease (NAFLD) shows epidemiological links to hypothyroidism, the genetic substrates of this relationship remain unresolved. Integrating large-scale genome-wide association studies with single-cell transcriptomics, spatial transcriptomics, and single-cell chromatin accessibility via state-of-the-art computational approaches, we interrogated the association between NAFLD and hypothyroidism across organ systems, cellular expression landscapes, and molecular–genetic strata. We uncovered pronounced spatial specificity in genetic risk within the liver, prioritized hepatocytes as the principal shared cell type affected, and, leveraging spatial transcriptomics, advanced a dynamic spatiotemporal two-hit model. We further nominated MAGI3, RRNAD1, and PRCC as high-confidence candidate genes and pinpointed a key risk locus, rs926103. These findings deliver a dynamic, testable framework for the full pathophysiological continuum linking NAFLD and hypothyroidism and yield new targets and leads for precision intervention.

## 1. Introduction

The human organism is intrinsically geared toward metabolic homeostasis; the metabolic–endocrine coupling of the hepato-thyroid axis not only underpins systemic equilibrium but also sustains growth and development, metabolic regulation, and overall physiological health [1,2]. From the vantage point of endocrine and metabolic balance, thyroid hormones broadly modulate hepatic lipogenesis, fatty acid oxidation, and cholesterol and bile acid metabolism [3,4], and help maintain hepatic homeostasis by inducing autophagy [5]. Clinically and at the population level, hepatic fat content and thyroid functional status are indispensable determinants of individual metabolism and systemic health [6].

In contemporary societies marked by caloric abundance and sedentary behavior, nonalcoholic fatty liver disease (NAFLD, now also termed as metabolic dysfunction-associated steatotic liver disease = MASLD [7])—characterized by hepatocellular steatosis, insulin resistance, and progressive inflammation–fibrosis—has become a leading cause of disability and metabolic burden worldwide, affecting roughly 24% of the global population [8]. At the liver level, it is accompanied by a series of changes, including hepatocellular lipid droplet accumulation with lipotoxicity, activation of hepatic stellate cells and fibrogenesis, persistent lobular inflammation, mitochondrial dysfunction with redox disequilibrium, and disruption of thyroid hormone signaling [9]. Despite advances in noninvasive diagnostics and therapeutics, the pathogenesis of NAFLD remains incompletely defined—particularly its interplay with hypothyroidism, which requires systems-level elucidation.

NAFLD encompasses a broad disease spectrum, ranging from simple steatosis to nonalcoholic steatohepatitis (NASH, now also termed as metabolic dysfunction-associated steatohepatitis = MASH), progressive fibrosis, and cirrhosis, and can ultimately culminate in hepatocellular carcinoma. Emerging evidence supports a “multiple-hit” model for NAFLD initiation and progression, in which genetic predisposition, diet-driven metabolic dysfunction, gut microbiome alterations, local immune reprogramming, and dysregulation of the spleen–liver axis act in concert [10]. Within this framework, hypothyroidism has been widely recognized as a key endocrine contributor that may both trigger NAFLD and accelerate its progression [11,12], thereby attracting substantial research interest. Stemming from insufficient thyroid hormone secretion or diminished peripheral action, hypothyroidism engenders a chronic hypometabolic state typified by a reduced basal metabolic rate, dysregulated lipid and glucose metabolism, dyslipidemia, and weight gain, often accompanied by long-standing autonomic dysfunction and immunometabolic imbalance [13,14]. Beyond lowering energy expenditure and elevating metabolic syndrome risk, thyroid hormone deficiency can trigger epigenetic alterations [15]; disrupt feedback within the hepato-thyroid axis and the hypothalamic–pituitary–thyroid (HPT) axis (e.g., altered hepatic deiodinase expression and attenuated TRβ signaling); promote inflammation and lipogenesis; impair fatty acid oxidation; and reduce hepatocellular lipophagy. Together, these processes forge a complex, bidirectional reinforcement with NAFLD [16,17]: hypothyroidism markedly increases the risk of NAFLD onset and progression, while persistent steatosis and inflammation can, in turn, impair thyroid hormone metabolism and axis feedback, exacerbating thyroid dysfunction and creating a difficult-to-break vicious cycle [18]. Clinically, NAFLD patients with concomitant hypothyroidism often display more severe phenotypes, poorer metabolic and antifibrotic treatment responses, higher risks of comorbidities such as cardiovascular disease, type 2 diabetes, and chronic kidney disease, and increased all-cause mortality [19].

Notably, the pathophysiology of hypothyroidism-induced NAFLD positions it as a potentially treatable and distinct disease entity [20]. In line with this premise, resmetirom, an oral thyroid hormone receptor-β (THR-β) agonist, has been approved for NASH [21], highlighting the therapeutic, screening, and early-intervention promise of the thyroid hormone/receptor (TH/TR) axis in NAFLD. Clarifying the mechanistic backdrop linking hypothyroidism and NAFLD is therefore essential both for deepening our understanding of comorbidity and for guiding the development of new interventions.

Progress stemming from the Human Genome Project and large-scale biobanks has substantially advanced our understanding of the genetic architecture of metabolic liver and endocrine diseases. Genome-wide association studies (GWASs) of NAFLD and thyroid function-related traits reveal partially overlapping loci, suggesting a shared genetic substrate [22,23]. Yet how these traits converge through expression and splicing quantitative trait loci (eQTLs and sQTLs) across human tissues; how they operate via specific cell types and interface with gene-regulatory networks and signaling pathways; and how these multilayered influences manifest as clinical heterogeneity remain insufficiently defined. Conventional single-layer approaches are ill-suited to decode such multiscale mechanisms, underscoring the need to integrate genetic data with multi-omic functional readouts.

To systematically delineate the NAFLD–hypothyroidism nexus, we integrated large-scale population genetic summary statistics with single-cell RNA sequencing (scRNA-seq), spatial transcriptomics, and single-cell chromatin accessibility, applying state-of-the-art cross-omic computational frameworks to interrogate shared and distinct biology across systems, cellular, and molecular layers. Specifically, we sought to identify shared risk loci, validate their tissue-level associations, resolve their regulatory effects across cell types, and define their concrete roles within convergent mechanisms, while charting the spatial expression of risk genes during embryonic development. This work aims to pinpoint the key molecules and functional linkages mediating the NAFLD–hypothyroidism interplay, thereby furnishing a rigorous foundation for targeted therapy, biomarker discovery, and early identification of high-risk populations. Ultimately, we anticipate that these insights will provide new perspectives and strategies for decoding the coupled mechanisms of NAFLD and hypothyroidism.

## 2. Results

### 2.1. Genome-Wide Meta-Analyses of NAFLD and Hypothyroidism

By meta-analyzing GWAS summary statistics from two independent cohorts per trait, we increased power to detect genetic associations for NAFLD and hypothyroidism. The NAFLD meta-analysis encompassed 10,924,601 genome-wide variants, and the hypothyroidism meta-analysis encompassed 10,694,841 variants.

### 2.2. Enrichment of NAFLD and Hypothyroidism Associations Within e/sQTLs

**Figure 1:**
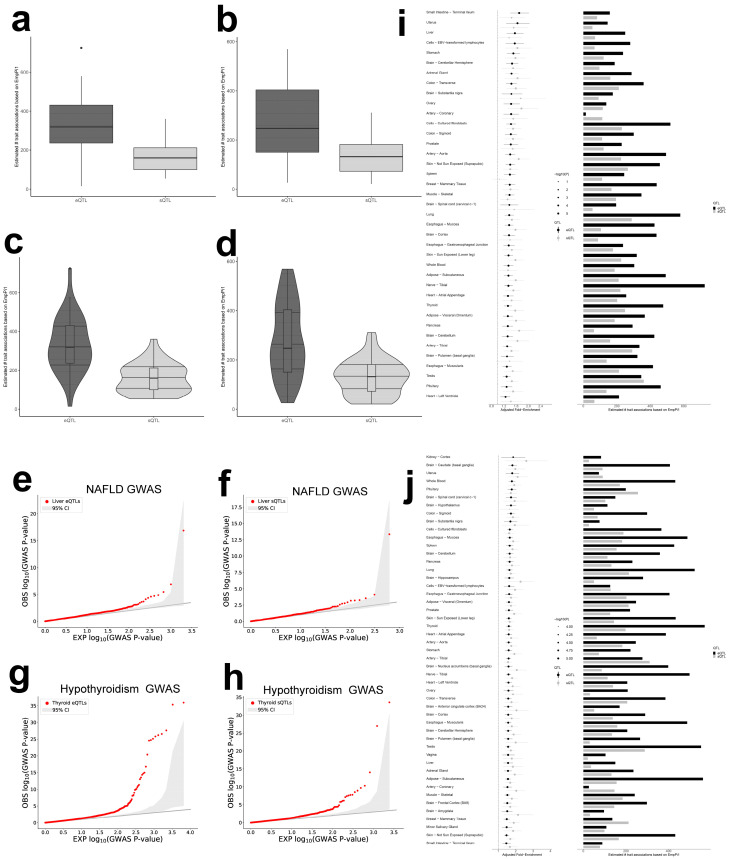
Boxplots showing the distribution of QTLEnrich statistics for NAFLD (**a**) and hypothyroidism (**b**), with eQTLs (dark gray) and sQTLs (light gray). Violin plots showing the distribution of QTLEnrich statistics for NAFLD (**c**) and hypothyroidism (**d**), with eQTLs (dark gray) and sQTLs (light gray). Q-Q plots of −log10 (*p*-value) of NAFLD GWAS for the significant best eQTL (**e**) or sQTL (**f**) per gene set (FDR < 0.05) in liver (red points): compared to GWAS *p*-values of 1000 confounder-matched null variant sets (light gray) generated by QTLEnrich. Q-Q plots of −log10 (*p*-value) of hypothyroidism GWAS for the significant best eQTL (**g**) or sQTL (**h**) per gene set (FDR < 0.05) in thyroid (red points): compared to GWAS *p*-values of 1000 confounder-matched null variant sets (light gray) generated by QTLEnrich. Forest plots (left panel) and bar plots (right panel) of all tissues whose eQTLs (dark gray) or sQTLs (light gray) were significantly enriched for NAFLD GWAS (**i**) or hypothyroidism GWAS (**j**) associations based on QTLEnrich. Points indicate the adjusted fold-enrichment scaled by −log10 (Enrichment *p*-value), and the lines represent 95% confidence intervals. Bar plots show the estimated number of e/sQTLs that are likely true trait associations per enriched tissue. NAFLD, nonalcoholic fatty liver disease; EmPi1, empirically estimated true positive rate of trait associations amongst significant best e/sQTLs per e/sGene (FDR < 0.05).

To assess organ-level relevance of regulatory variation, we applied QTLEnrich and validated the significance and robustness of e/sQTL enrichments (Figure 1a–h). Across 49 GTEx v8 tissues, both eQTLs and sQTLs were significantly enriched among trait associations for NAFLD and hypothyroidism (*p* < 0.005; Supplementary Table S1). Considering Adjusted_Fold_Enrichment together with the estimated number of trait associations with regulatory effects, the liver and thyroid emerged as principal sites of cumulative involvement (Figure 1i,j).

### 2.3. MAGMA Enrichment and Spatial Mapping Delineate Tissue-Specific Architecture

**Figure 2:**
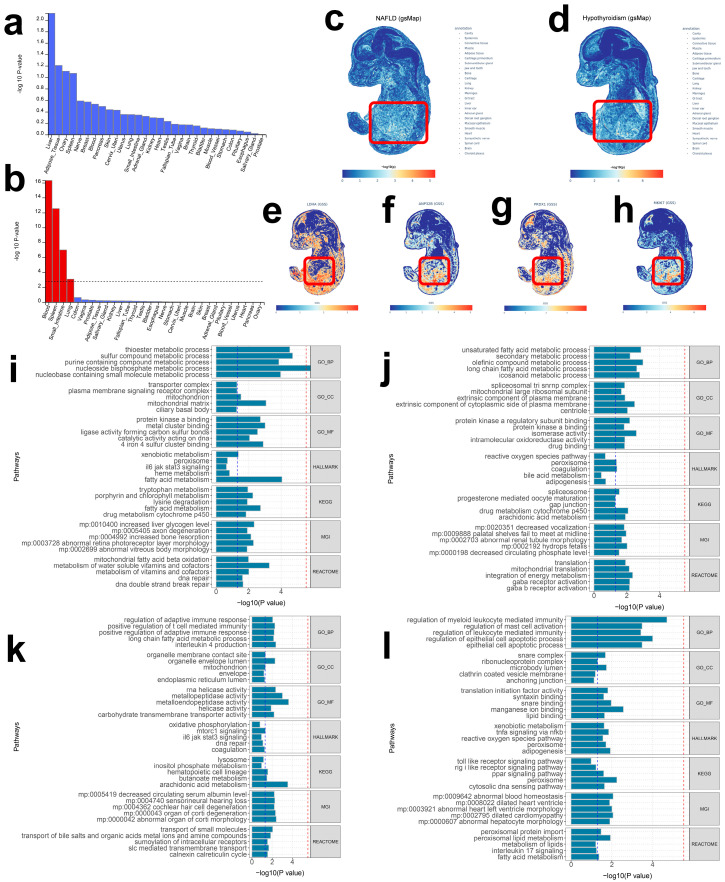
The bar graph shows the results of enrichment of tissues associated with NAFLD (**a**) and hypothyroidism (**b**) using MAGMA. The horizontal axis represents different tissues, and the vertical axis represents −log10 (*p*-value). GWAS genetic signals for NAFLD (**c**) and hypothyroidism (**d**) mapped to the spatial transcriptome landscape of E16.5 mouse embryonic tissues. Genes associated with hypothyroidism GWAS data, including LDHA (**e**), ANP32B (**f**), PRDX1 (**g**), and MKI67 (**h**), are significantly mapped in the liver. eGenes (**i**) and sGenes (**j**) significantly associated with NAFLD were significantly enriched in multiple gene sets in liver tissue; eGenes (**k**) and sGenes (**l**) significantly associated with hypothyroidism were significantly enriched in multiple gene sets in liver tissue. The horizontal axis represents −log10 (*p*-value), with the blue dashed line at *p* = 0.05, the red dashed line at *p* = 1 × 10^−5^. The vertical axis represents pathways.

To refine tissue specificity, we conducted MAGMA-based gene-level enrichment. Signals for both NAFLD and hypothyroidism were markedly enriched in liver and blood (*p* < 0.05; Figure 2a,b; Supplementary Table S2), consistent with thyroid hormone transport and downstream effects on glucose and lipid metabolism. Using gsMap, we projected GWAS signals onto an E16.5 mouse embryo spatial transcriptomic atlas and observed strong hepatic localization for both traits (Figure 2c,d). Notably, hypothyroidism-associated genes also exhibited pronounced hepatic mapping (Figure 2e–h), highlighting a spatial route through the liver that may underlie disease interplay.

### 2.4. Functional Enrichment Reveals Liver-Centric Biological Programs

We next used GeneEnrich to interrogate functional over-representation of trait-implicated genes, focusing on the liver given the tissue enrichments (Supplementary Table S3). Hypergeometric and permutation tests identified significant enrichment across multiple gene sets for both traits (Figure 2i–l). In the liver, NAFLD eQTLs were enriched for thioester metabolic process (GO_BP, empirical *p* < 0.05), fatty acid metabolism (HALLMARK/KEGG, empirical *p* < 0.05), increased liver glycogen level (MGI, empirical *p* < 0.05), and mitochondrial fatty acid β-oxidation (REACTOME, empirical *p* < 0.05). NAFLD sQTLs were enriched for unsaturated fatty acid metabolic process (GO_BP, empirical *p* < 0.05), long-chain fatty acid metabolic process (GO_BP, empirical *p* < 0.05), arachidonic acid metabolism (KEGG, empirical *p* < 0.05), and integration of energy metabolism (REACTOME, empirical *p* < 0.05). For hypothyroidism, liver eQTLs were enriched for long-chain fatty acid metabolic process (GO_BP, empirical *p* < 0.05), carbohydrate transmembrane transporter activity (GO_MF, empirical *p* < 0.05), arachidonic acid metabolism (KEGG, empirical *p* < 0.05), and transport of small molecules (REACTOME, empirical *p* < 0.05); sQTLs were enriched for lipid binding (GO_MF, empirical *p* < 0.05), adipogenesis (HALLMARK, empirical *p* < 0.05), abnormal hepatocyte morphology (MGI, empirical *p* < 0.05), and fatty acid metabolism (REACTOME, empirical *p* < 0.05). These findings suggest partially divergent biological routes in the hepatic compartment for the two traits.

NAFLD, nonalcoholic fatty liver disease; GO, Gene Ontology; BP, Biological Processes; MF, Molecular Function; CC, Cellular Components; KEGG, Kyoto Encyclopedia of Genes and Genomes; MGI, Mouse Genome Informatics.

### 2.5. Cellular Composition in NAFLD and Hypothyroidism

**Figure 3:**
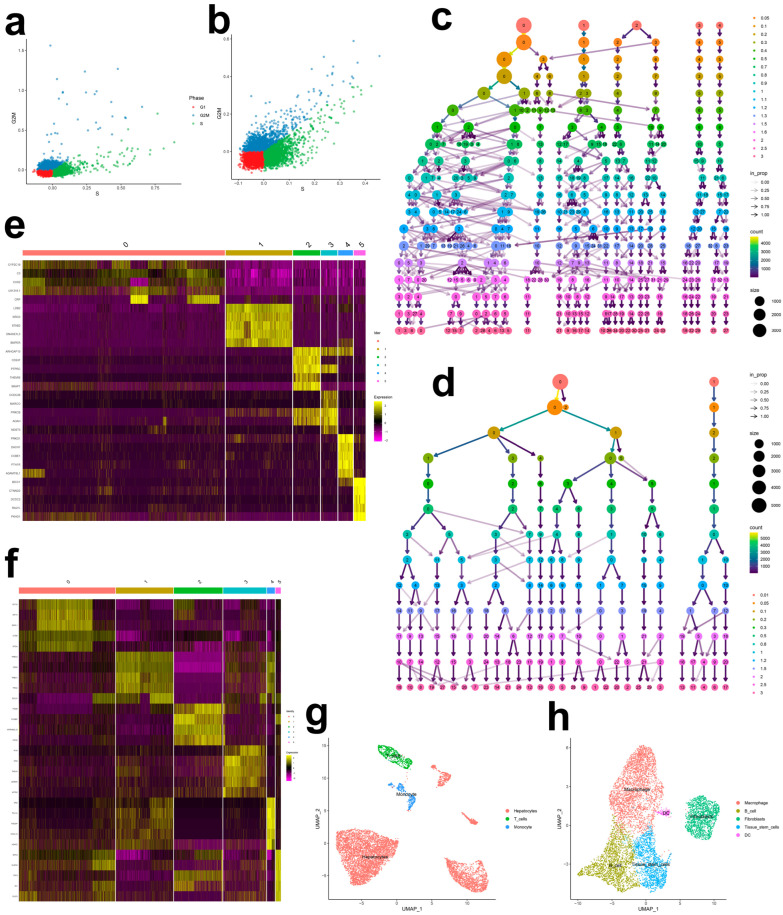
Cell cycle distribution diagrams of single-cell transcriptome data for NAFLD (**a**) and hypothyroidism (**b**), with red dots indicating cells in the G1 phase, blue dots indicating cells in the G2/M phase, and green dots indicating cells in the S phase. Cluster dendrograms of single-cell transcriptome data at different resolutions for NAFLD (**c**) and hypothyroidism (**d**). Heatmap of marker genes significantly overexpressed in different clusters of single-cell transcriptome data for NAFLD (**e**) and hypothyroidism (**f**). UMAP plots of cell-type annotation of single-cell transcriptome data for NAFLD (**g**) and hypothyroidism (**h**).

Following stringent QC, normalization, and Harmony integration (Supplementary Figure S1a–d), we performed unsupervised clustering and cell-type identification. Elbow plots supported dimensionality reduction quality (Supplementary Figure S1e,f), and t-SNE on Harmony embeddings showed effective batch correction and clear biological structure (Supplementary Figure S1g,h). Cell-cycle scoring (S and G2M) revealed marked heterogeneity with cycle-active subpopulations (Figure 3a,b). Using Harmony embeddings, we built neighborhood graphs and conducted multiresolution clustering (13 resolutions spanning 0.01–3.0), selecting stable partitions via a clustering tree (Figure 3c,d). Optimal resolutions were 0.05 for NAFLD and 0.2 for hypothyroidism. Marker genes per cluster informed heatmaps (Figure 3e,f), and SingleR-enabled annotation identified hepatocytes, T cells, and monocytes in NAFLD, and macrophages, B cells, fibroblasts, tissue stem cells, and dendritic cells in hypothyroidism (Figure 3g,h).

### 2.6. Cell-Type Signals from ECLIPSER

ECLIPSER revealed significant cell-type-specific enrichment for both traits (Table 1). NAFLD loci were most enriched in monocytes (fold-enrichment = 7.61; 95% CI, 1.33–16.27; enrichment *p* < 0.05), while hypothyroidism risk was localized to fibroblasts (fold-enrichment = 2.58; 95% CI, 0.80–5.65; enrichment *p* < 0.05), indicating potential roles for these cell types in disease processes.

### 2.7. CELLECT Prioritizes Hepatocytes and Immune Compartments

Using S-LDSC within CELLECT, NAFLD heritability was significantly enriched in hepatic hepatocytes (*p* < 0.05), whereas hypothyroidism heritability was enriched in liver-resident natural killer cells (*p* < 0.05) and other immune subsets. MAGMA gene-set analyses corroborated and extended these findings: hepatocyte-specific expression sets were associated with NAFLD (*p* < 0.05), and natural killer and B-cell expression sets in liver were associated with hypothyroidism (*p* < 0.05; Supplementary Table S4).

### 2.8. Integrative Cell-Type Prioritization

We integrated three orthogonal lines of evidence—single-cell atlas annotation, ECLIPSER, and CELLECT—to prioritize disease-relevant cell types, assigning one point per supporting modality (Table 2). Hepatocytes attained the highest cumulative score (3), underscoring their centrality, and immune populations in the liver and thyroid received elevated scores, implicating immunity in disease progression.

### 2.9. Candidate Gene Networks in Hepatocytes

**Figure 4:**
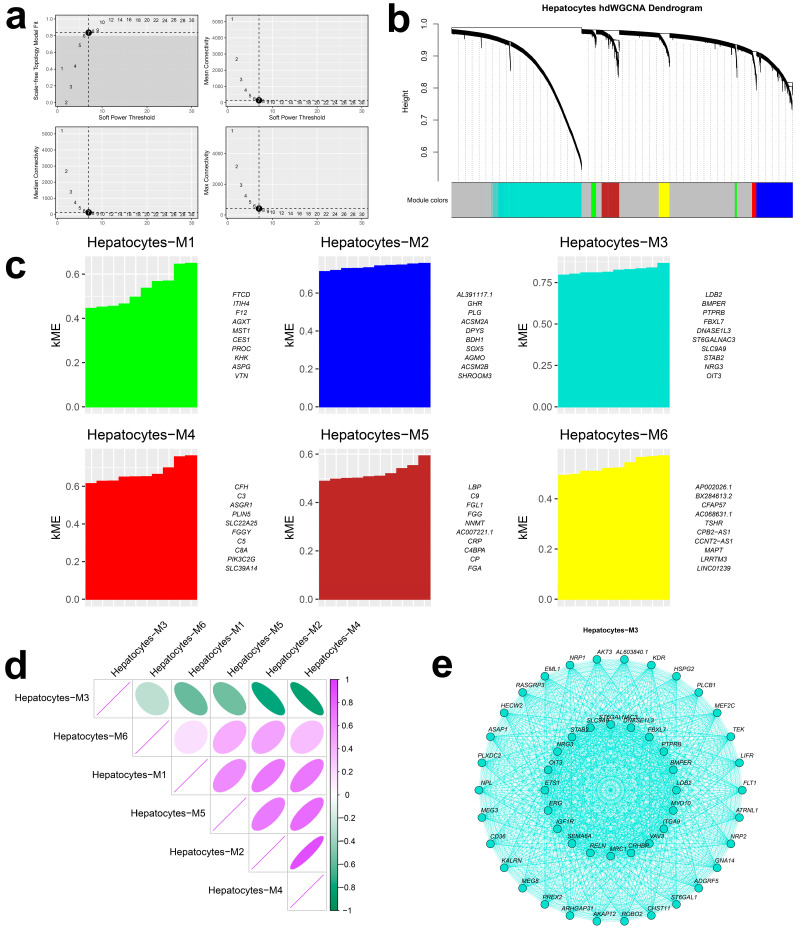
(**a**) Analysis of network topology for various soft-thresholding powers. (**b**) Construction of co-expression network based on optimal soft threshold, dendrogram of different gene modules. (**c**) Module eigengenes of different gene modules. (**d**) Correlation heatmap of different gene modules; purple indicates positive correlation and green indicates negative correlation. (**e**) Gene network diagram of the top 50 genes in the M3 gene module. kME, Module Eigengene-based Connectivity.

Given hepatocyte prioritization, we applied hdWGCNA to delineate transcriptional programs within this lineage. To mitigate sparsity, metacells were constructed (k = 25). A soft-threshold power of 7 was selected (Figure 4a), and a signed topological overlap network was assembled to approximate scale-free topology. Dynamic tree cutting identified six co-expression modules (Figure 4b,c). Notably, module M3 was broadly anticorrelated with the remaining modules (Figure 4d). Candidate regulators within M3 included LDB2, BMPER, PTPRB, FBXL7, DNASE1L3, ST6GALNAC3, SLC9A9, STAB2, NRG3, and OIT3 (Figure 4e).

### 2.10. Colocalization with eCAVIAR

Bayesian colocalization pinpointed putative causal genes and regulatory variants shared with GWAS loci. We identified 41 loci (218 genes) colocalizing with NAFLD (CLPP > 0.01) and 118 loci (531 genes) colocalizing with hypothyroidism (CLPP > 0.01), with enrichment in the liver, thyroid, and blood. Exemplars included LTB4R eQTLs colocalizing with NAFLD risk in the liver (CLPP = 0.82), CPN1 sQTLs colocalizing with NAFLD in the liver (CLPP = 0.86), RRNAD1 eQTLs colocalizing with hypothyroidism in the thyroid (CLPP = 1), and MAGI3 sQTLs colocalizing with hypothyroidism in the thyroid (CLPP = 1).

### 2.11. Colocalization with fastENLOC

**Figure 5:**
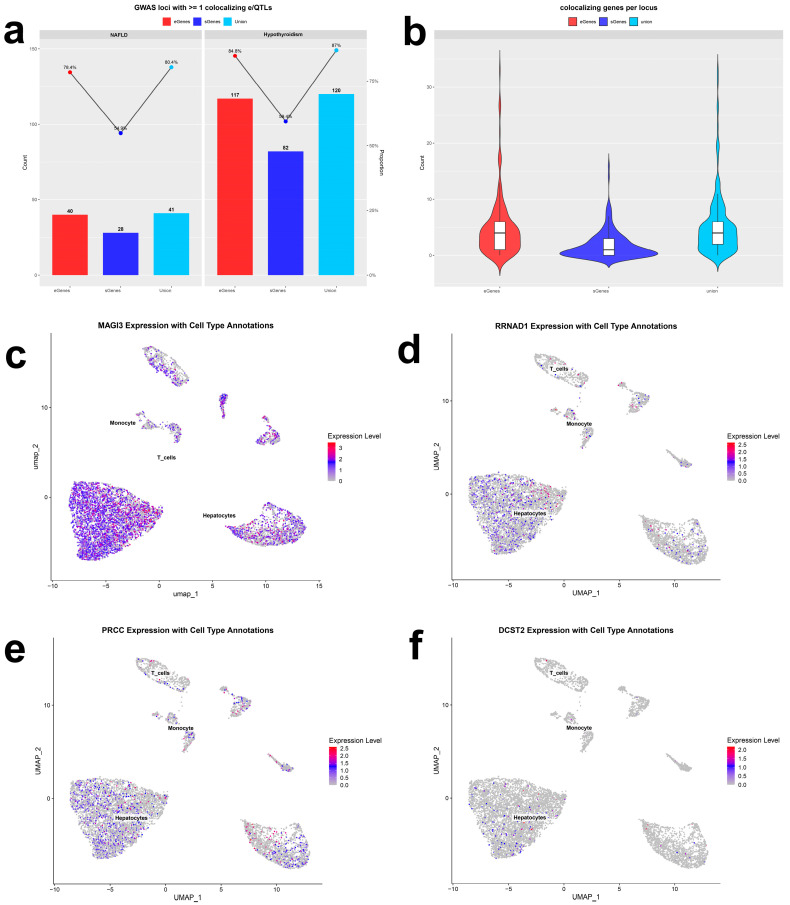
(**a**) Histogram of percent of GWAS loci with ≥1 colocalizing e/sQTL (CLPP > 0.01 from eCAVIAR and/or RCP >  0.1 from fastenloc) for NAFLD and hypothyroidism GWAS meta-analyses. Numbers above the bars represent the number of loci with at least one colocalizing e/sQTL. Red, dark blue, and light blue bars indicate the percentage of loci with at least one colocalizing eGene, sGene, or both, respectively. Union, gene with at least one significant e/sQTL. (**b**) Violin plots showing the distribution of the unique number of colocalizing eGenes (red), sGenes (dark blue), or both (light blue) per locus for the two GWAS meta-analyses tested. The center line in the box plots contained within each violin plot shows the median; the box edges depict the interquartile range (IQR). The violin plot edges represent the minima and maxima values. Expression of MAGI3 (**c**), RRNAD1 (**d**), PRCC (**e**), and DCST2 (**f**) genes in different cells of NAFLD single-cell transcriptome data. The color red indicates higher expression. CLPP, colocalization posterior probability; RCP, regional colocalization probability.

Using fastENLOC across 49 GTEx v8 tissues, we found 34 loci (254 genes) colocalizing with NAFLD (RCP > 0.1) and 38 loci (215 genes) colocalizing with hypothyroidism (RCP > 0.1). Highlights included RSG1 eQTLs colocalizing with NAFLD risk in the liver (RCP = 0.99), HSD17B13 sQTLs colocalizing with NAFLD in the liver (RCP = 0.99), RRNAD1 eQTLs colocalizing with hypothyroidism in the thyroid (RCP = 0.95), and MAGI2 sQTLs colocalizing with hypothyroidism in the thyroid (RCP = 0.96). Collectively, we identified 41 and 120 e/sQTL colocalizations for NAFLD and hypothyroidism, respectively (CLPP > 0.01 and/or RCP > 0.1; Figure 5a,b; Supplementary Table S5).

### 2.12. Open4Gene Hurdle Analysis

Across immune cell types, Open4Gene identified 17,076 significant cis peak–gene regulatory pairs (hurdle zero-part *p* > 0.05 and count-part *p* < 0.05), involving 6816 genes (Supplementary Table S6).

### 2.13. Expression Annotation and Exon-Level Splicing of Prioritized Genes

**Figure 6:**
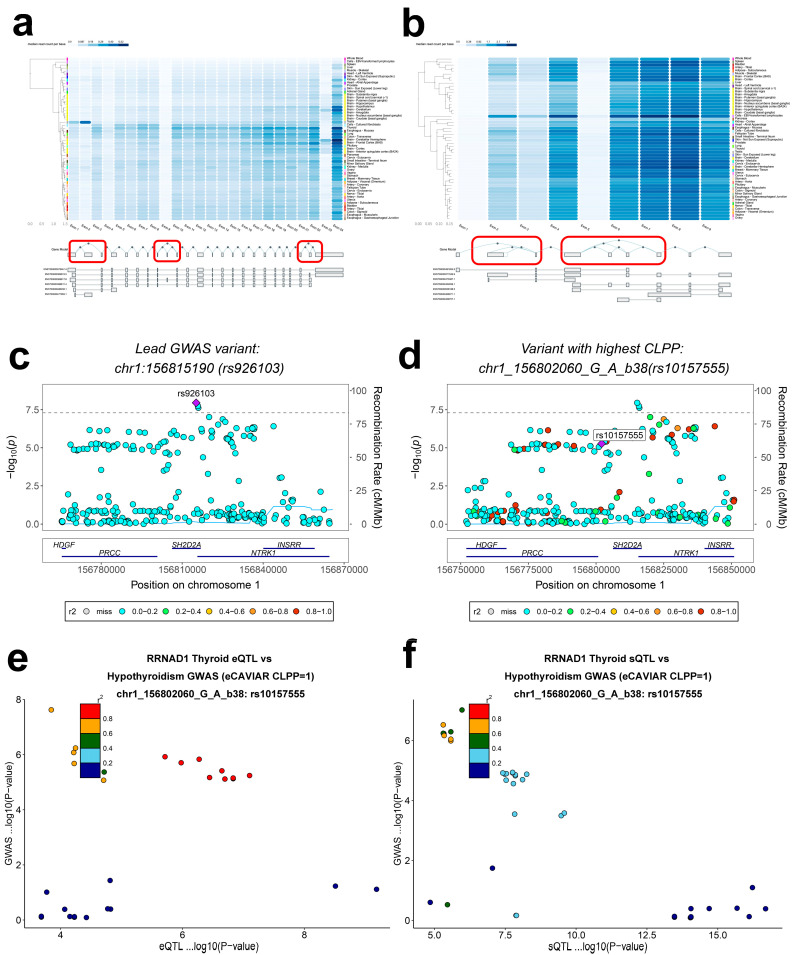
Exon boxes in MAGI3 (**a**) and PRCC (**b**) gene models. Connecting lines represent all splicing events between exons. The heat map above shows the expression level of the gene in different tissues. The darker the blue, the higher the expression level. Plots are taken from https://gtexportal.org/ (accessed on 10 October 2025). (**c**) LocusZoom plot of the colocalization position of rs926103 on chromosome 1. (**d**) LocusZoom plot of the colocalization position of rs10157555, which is most closely related to rs926103, on chromosome 1. LocusCompare plots of hypothyroidism GWAS meta-analysis −log10 (*p*-value) versus −log10 (*p*-value) of RRNAD1 eQTL (**e**) and sQTL (**f**) in the thyroid. Darker dots indicate larger r^2^.

Integrating multiple prioritization criteria (Table 3), we nominated four high-confidence genes—MAGI3, RRNAD1, PRCC, and DCST2. UMAP overlays showed higher expression of MAGI3, RRNAD1, and PRCC in hepatocytes, with DCST2 being the lowest in this lineage (Figure 5c–f). Exon-level profiling revealed that MAGI3 is highly expressed in the thyroid relative to other tissues, potentially reflecting tissue-specific alternative splicing across exons 1–3, 8–10, and 22–24 (Figure 6a). PRCC showed broad expression across tissues, including liver and thyroid, with exon 1–7 splicing events plausibly contributing to inter-tissue differences (Figure 6b)

### 2.14. Genomic Risk Loci

We identified 189 independent genome-wide significant SNPs distributed across the genome, with notable enrichment on chromosome 1. Colocalization highlighted a high-risk locus, rs926103, whose interval encompassed PRCC (Figure 6c) [24] and implicated both PRCC and RRNAD1 (Supplementary Table S5). rs10157555 was highly correlated with rs926103 (Figure 6d); its eQTL and sQTL linked RRNAD1 to hypothyroidism (Figure 6e,f). GCTA-COJO conditional analysis within the rs926103 region showed that rs10157555 was not significant after conditioning (*P*_conditional = 0.73), suggesting dependence on rs926103, whereas rs55700401 and rs114551306 remained significant (*P*_conditional < 0.05), underscoring the central role of rs926103 in the shared pathogenesis of NAFLD and hypothyroidism (Supplementary Table S7).

## 3. Discussion

NAFLD is a chronic liver disorder and the hepatic manifestation of metabolic syndrome; its pathological ties and shared genetic underpinnings with metabolic diseases have long been a focal point of inquiry. The etiology is multifactorial, reflecting multiple, parallel “hits” wherein genetic susceptibility intersects environmental/metabolic exposures—insulin resistance, shifts in the gut microbiota, immune–inflammatory activation, and oxidative stress [25,26]. Large-scale genomics has implicated several loci with robust associations to NAFLD risk, including PNPLA3 (rs738409), ABCC8 (rs146378237), and ApoC-III (rs2854116, rs2854117), suggesting that compounded genetic risk may exacerbate disease severity [27,28,29,30]. Yet converting statistical associations into testable and actionable biology remains a central bottleneck, underscoring the need for cross-omics functional annotation. Here we seek to bridge this gap: rather than cataloging additional associations, we integrate a large-scale GWAS meta-analysis, multi-tissue regulatory maps (QTL), single-cell liver atlases, and spatial transcriptomics to assemble a mechanism-oriented framework from systems to cells to molecules. Our analyses indicate that the genetic architecture of NAFLD and its salient risk factor, hypothyroidism, is nonrandom—converging on specific cell types, molecular circuits, and developmental windows. We organize the discussion around four principal insights: (1) tissue-specific convergence of genetic risk underlies hepatic lipid dysregulation; (2) hepatocytes act as central integrators, linking genetic, immune, and endocrine stress through lipid-droplet mobilization and metabolic networks; (3) the developmental-origin hypothesis gains genetic support, motivating a spatiotemporally dynamic disease model; and (4) high-confidence candidates—MAGI3, RRNAD1, and PRCC—and the risk locus rs926103 nominate tractable targets for precision therapeutics.

A signature result is the pronounced spatial specificity of genetic risk across the liver for both NAFLD and hypothyroidism. Thyroid hormones orchestrate thermogenesis, adipogenesis and its distribution, and broader energy metabolism [31]. Within the hypothalamic–pituitary–thyroid (HPT) axis, thyroid hormones circulate predominantly as triiodothyronine (T3), the biologically active form, and thyroxine (T4), which functions largely as a prohormone. T4 is converted to T3 both systemically and within target tissues by iodothyronine deiodinases; notably, type 2 deiodinase (D2) catalyzes the activation of T4 to T3. Thyroid hormones promote lipolysis in white adipose tissue (WAT) and facilitate the mobilization of dietary lipids, thereby increasing circulating free fatty acids (FFAs), which constitute a major substrate pool for hepatic lipid accumulation. FFAs are imported into hepatocytes through fatty acid transport proteins (FATPs), liver-type fatty acid-binding proteins (L-FABPs), and the fatty acid translocase CD36 (also referred to as FAT). Concurrently, thyroid hormones enhance intrahepatic lipid utilization by stimulating hepatic lipolysis and fatty-acid metabolic pathways (including β-oxidation), thereby supporting the clearance of excess lipids and contributing to the attenuation of hepatic steatosis [32]. The receptors THR-α1 and THR-β1 constitute the principal isoforms [33]; THR-β is predominantly expressed in liver and ventricular myocardium and is downregulated in NASH [34]. Moreover, 3,5-diiodothyronine (T2) reduces obesity and dyslipidemia and reverses steatosis [35], likely by modulating hepatic lipase activity to accelerate lipid-droplet mobilization [36]. Conversely, elevated TSH—a hallmark of hypothyroidism—promotes hepatic lipogenesis and gluconeogenesis [37]. By engaging the TSH receptor, TSH augments hepatocellular triglyceride uptake and fosters steatosis; TSHR activates SREBP-1c via the cAMP/PKA/PPARα axis while attenuating AMPK, collectively driving lipogenesis [38]. These observations cement the liver’s foundational role in the shared pathophysiology of NAFLD and hypothyroidism. Beyond direct effects, the thyroid shapes hepatic lipid metabolism through central circuits [39]. Thyroid hormone suppresses PCSK9 and lowers low-density lipoprotein (LDL); hypothyroidism correlates with elevated PCSK9 [40], predisposing to hepatic lipid accumulation. In parallel, thyroid hormone modulates carbohydrate-responsive element-binding protein (ChREBP) and Liver X Receptor (LXR), indirectly driving transcriptional programs of hepatic lipogenesis [41], highlighting the thyroid–liver axis in disease evolution. Functionally, our gene-set analyses reveal marked enrichment of lipid synthetic and catabolic programs—including fatty acid metabolism—within genetic risk, implicating energy-metabolic derangement as a core mechanism and aligning with prior evidence. Complementarily, hypothyroidism-associated signals were enriched not only for lipogenesis and fatty-acid metabolism overlapping NAFLD risk, but also for pathways linked to abnormal hepatocyte morphology, consistent with the substrate for progression to cirrhosis and carcinoma and with PNPLA3 risk-associated hepatocellular injury [29].

Hepatocytes account for approximately 70–80% of liver volume and execute essential metabolic programs [42]. In our analyses, hepatocytes emerged as the top shared vulnerable cell type across NAFLD and hypothyroidism—shifting the lens from “passive droplet accumulation” to “active metabolic control.” Hepatocytes generate energy via β-oxidation and buffer surplus free fatty acids or glucose by channeling them into triglyceride-rich lipid droplets, thereby maintaining lipid homeostasis [43]. They also clear excess droplets through lipophagy, a cornerstone of adult hepatic equilibrium and adaptation to nutritional flux; genetic risk and metabolic state may perturb this program, yielding net droplet accumulation and lipotoxicity [43,44]. Beyond droplet metabolism, hepatocytes coordinate with stellate cells and liver macrophages in spatial cross-cell networks, integrating genetic and environmental stressors at the tissue scale to shape disease trajectories [45]. hdWGCNA further identified highly connected hepatocyte modules enriched for energy metabolism, suggesting that genetic risk primes these cells for lipid-homeostatic failure under endocrine stress.

Our data support a developmental-origin model. Projecting GWAS signals onto embryonic mouse liver spatial atlases revealed that zonated, lipid-metabolism programs are established in utero [46], providing a molecular substrate for early “vulnerability setting.” We thus propose a spatiotemporal two-hit model: the first hit arises during development, whereby genetic risk specifies inherent fragility within distinct metabolic zones; the second unfolds later in life, when endocrine and metabolic stressors (e.g., hypothyroidism) amplify pre-existing zonal liabilities, escalating lipid-droplet disequilibrium and inflammation–fibrosis cascades and culminating in organ dysfunction.

Gene prioritization converged on MAGI3, RRNAD1, and PRCC as high-confidence candidates, each showing pronounced colocalization in thyroid and strong expression in hepatocytes. Prior genomics implicates MAGI3 in both NAFLD and hypothyroidism [47,48], nominating it as a plausible substrate for comorbidity. As a tight-junction scaffolding protein, MAGI3 preserves intercellular architecture [49]; it can suppress Wnt/β-catenin signaling [50], compromise intestinal barrier integrity, perturb the gut–liver axis, and thereby drive NAFLD progression [51,52]. PRCC, a component of the pre-mRNA splicing apparatus, shows inverse relationships with DNA damage–response mediators, including ATM/ATR and HIPK2 [53]; given that DNA synthesis and repair pathways are candidate drivers in NAFLD [54], PRCC may modulate disease by influencing damage and repair dynamics. Moreover, tissue-specific alternative splicing of MAGI3 and PRCC may represent shared post-transcriptional liabilities in NAFLD and hypothyroidism, broadening the genetic landscape of their molecular etiology.

Focusing on genomic risk and conditional signals, we highlight rs926103 alongside two independent secondary associations, rs55700401 and rs114551306. rs926103 resides within the open reading frame of SH2D2A, which encodes the adaptor TSAd. Although TSAd lacks catalytic domains, it acts as a regulator and transcriptional activator across signaling pathways, modulating tyrosine kinase activity and downstream cascades [55], suggesting that interactions with regulatory elements may influence hepatocellular metabolic circuitry.

In aggregate, our findings indicate that genetic risk converges spatially within the liver and, when compounded by hypothyroidism-mediated control of energy metabolism, manifests as aberrant hepatocellular lipid handling and steatosis. Multiscale integration across regulatory genetics, single-cell, and spatial dimensions anchors statistical signals to the hepatic microenvironment, specific lineages, and discrete loci, providing coordinates for precise target nomination. These hypotheses warrant validation in hepatocyte models to delineate effects on energy metabolism, lipid-droplet mobilization, and post-transcriptional regulation.

Notwithstanding these advances, limitations remain. eQTL/sQTL resources largely derive from postmortem healthy tissues and do not capture dynamic regulation under disease, necessitating validation in patient-derived samples and human in vitro systems. Because thyroid biopsy is not clinically indicated for hypothyroidism, suitable single-cell RNA-seq datasets are scarce; we therefore used Hashimoto’s thyroiditis as a proxy, which does not encompass other etiologies, underscoring the need for dedicated single-cell profiling. Importantly, the present study is inherently computational and hypothesis-generating. While the GWAS meta-analysis, QTL integration, and colocalization analyses strengthen biological plausibility, the nominated genes, regulatory mechanisms, and loci should be interpreted as predictive rather than definitive evidence of causality. In particular, colocalization and fine-mapping are constrained by linkage disequilibrium structure and model assumptions and cannot fully exclude correlated signals or horizontal pleiotropy. Finally, NAFLD and hypothyroidism exhibit regional and ancestral heterogeneity; large, multi-ancestry cohorts will be essential to assess the specificity and transferability of genetic risk.

## 4. Methods and Materials

### 4.1. Sources of Genome-Wide Summary Statistics

We analyzed GWAS summary statistics for NAFLD and hypothyroidism, each aggregated from two independent cohorts. The NAFLD GWAS included 500,348 and 377,988 participants, and the hypothyroidism GWAS included 406,300 and 8802 participants, each comprising cases and controls. Across studies, genotype–phenotype associations were leveraged to identify disease-associated single-nucleotide polymorphisms (SNPs).

### 4.2. Single-Cell Multi-Omics Data from Peripheral Blood Mononuclear Cell (PBMC) from a Healthy Donor

PBMCs from a healthy 25-year-old female donor were profiled using the 10× Genomics Chromium Single Cell Multiome platform. Following flow cytometric depletion of granulocytes, nuclei were isolated as per the 10× standard protocol (CG000365 Rev A), yielding 11,909 high-quality cells. Paired Assay for Transposase Accessible Chromatin with high-throughput sequencing (ATAC-seq) and gene expression libraries were prepared with Chromium Next GEM Single Cell Multiome reagents (CG000338 Rev A, Cellenion, Lyon, France) and sequenced on an Illumina NovaSeq 6000 (Illumina, San Diego, CA, USA) using a stranded dual-index strategy (gene expression cycles: 28–10–10–90; ATAC cycles: 50–8–16–49). The resulting dataset comprised a median of 1826 genes and 3776 UMIs per cell for expression and 13,486 high-quality fragments per cell for chromatin accessibility, encompassing 108,377 open chromatin peaks and 15,494 genes, with 85,468 peak–gene links resolved.

### 4.3. Single-Cell Transcriptomic Datasets

For NAFLD, we curated publicly available single-cell datasets generated from liver biopsies of patients diagnosed with NAFLD, NASH, or NASH with cirrhosis, including transplant samples from end-stage disease, alongside healthy donor livers as controls (GSE202379). Tissue was snap-frozen in liquid nitrogen, nuclei were isolated via tissue lysis and FACS purification, and libraries were prepared on the 10× Genomics platform. Given the lack of clinical indication for thyroid biopsy in hypothyroidism, we used single-cell datasets from Hashimoto’s thyroiditis—the predominant cause of hypothyroidism [56]—including lesional and adjacent normal thyroid tissues collected at the Surgical Department of Hospital Universitario La Princesa (GSE248205). Thyroid samples were snap-frozen in liquid nitrogen pre-cooled with isopentane, stored at −80 °C, RNA was extracted from freshly frozen tissue, and libraries were prepared on the 10× Genomics platform. For downstream analyses focused on NAFLD and hypothyroidism, we included only the NAFLD vs. control liver biopsies and Hashimoto’s thyroiditis vs. control thyroid samples.

### 4.4. Quality Control

GWAS Quality control (QC) included: (i) filtering variants with minor allele frequency (MAF) < 0.01; (ii) harmonizing genome build across datasets; and (iii) excluding SNPs within the MHC region (chromosome 6) from hypothyroidism GWAS due to extensive linkage disequilibrium (LD) and the autoimmune etiology of hypothyroidism. For scRNA-seq, we used Seurat for preprocessing and computed per-cell proportions of mitochondrial genes (prefix “^MT-“) and hemoglobin genes (e.g., HBA1, HBA2, HBB). We retained high-quality cells with UMIs ≥ 1000, 200–5000 detected genes, mitochondrial content ≤ 15%, and hemoglobin content ≤ 3%, visualized via violin plots pre- and post-QC. Dimensionality reduction used PCA and t-SNE, with heatmaps and elbow plots for quality assessment. Batch effects were mitigated using Harmony (version 0.1) (theta = 2, lambda = 1) in R (version 4.4.3).

### 4.5. Genome-Wide Meta-Analysis

To enhance statistical power, we performed fixed-effects meta-analyses, integrating GWAS summary statistics from two independent cohorts per trait to increase sensitivity for detecting small-effect variants.

### 4.6. QTLEnrich: Tissue-Specific e/sQTL Enrichment for NAFLD and Hypothyroidism

We assessed tissue-level relevance across 49 GTEx v8 tissues by testing enrichment of trait associations among eQTLs and sQTLs using QTLEnrich, a rank- and permutation-based framework that accounts for MAF, distance to transcription start site, and local LD. Adjusted_Fold_Enrichment and Enrichment_*p*_value were used to quantify significance. We further generated QQ plots comparing observed versus theoretical −log10(*p*) to visualize enrichment.

### 4.7. Tissue Enrichment via MAGMA

Complementing QTLEnrich, we performed MAGMA-based gene-level enrichment to identify tissues associated with NAFLD and hypothyroidism. GWAS summary data were formatted for MAGMA, and tissues with *p* < 0.01 were deemed credible.

### 4.8. Spatial Mapping of Trait-Relevant Cells at Single-Cell Resolution

To chart spatial specificity for NAFLD and hypothyroidism, we integrated single-cell spatial transcriptomics (sc-ST) with GWAS statistics using genetically informed spatial mapping (gsMap) of cells for complex traits. This cross-species framework integrates mouse embryonic, macaque cortex, and human GWASs to infer spatial distributions of trait-relevant cellular populations by projecting GWAS-derived trait genes onto spatially resolved cells. Using the E16.5 mouse embryo spatial atlas (25 organs), we generated spatial enrichment maps and spatial expression maps for trait genes, assembling a single-cell–resolution spatial pathomechanistic atlas.

### 4.9. Functional Enrichment with Gene Enrich

We applied GeneEnrich to evaluate over-representation of candidate gene sets in curated functional/phenotypic collections using hypergeometric and permutation tests. To reduce tissue-specific bias, empirical *p*-values were obtained by permutation. Functional categories were sourced from Gene Ontology (GO) [57], Reactome, Kyoto Encyclopedia of Genes and Genomes (KEGG) [58], Molecular Signatures Database (MSigDB), and Mouse Genome Informatics (MGI). Nominal significance was defined as empirical *p* < 0.05.

### 4.10. Annotation of Single-Cell Atlases to Identify Disease-Relevant Cell Signals

Using Harmony-corrected embeddings, we visualized data via principal component heatmaps, elbow plots, t-SNE, and Harmony projections. Neighborhood graphs were constructed on Harmony embeddings, and clustering was performed across 13 resolutions (0.01–3.0). An optimal resolution was selected from the clustering tree to yield stable subpopulations. Marker genes per cluster were identified with FindAllMarkers (min.pct = 0.25, log_2_FC threshold = 0.25), and marker-based heatmaps were generated. Cell types were annotated using SingleR for automated labeling.

### 4.11. Identifying Cell-Type Signals with ECLIPSER

ECLIPSER [59,60] estimated cell-type–specific enrichment for each trait–tissue–cell-type combination using Bayesian Fisher’s exact framework, benchmarking against background GWAS loci. Cell-type specificity thresholds were set at the 95th percentile of background locus scores, and 95% confidence intervals for fold-enrichment were reported to enable robust inference with sparse signals. LD expansion of index loci used r^2^ > 0.8 to capture proxy variants. For differential expression within each cell type, we applied Wilcoxon tests (minimum cells per group = 3; gene min.pct = 10%; log_2_FC threshold = 0.5; adjusted *p* < 0.05). Cell types with *p* ≤ 0.05 were considered significantly enriched.

### 4.12. CELLECT: Partitioning Heritability and Gene-Level Associations by Cell Type

We leveraged CELLECT to quantify the contribution of cell-type-specific expression to trait heritability using two complementary methods: stratified LD score regression (S-LDSC) [61] and MAGMA gene analysis [62]. The baseline cell-type reference was the Tabula Muris dataset. LD scores were computed using European samples from 1000 Genomes Project Phase 3. MAGMA assessed correlations between gene-level association statistics and average expression per cell type and performed pairwise conditioning to evaluate independence from other informative cell types. Significance for both methods was set at *p* < 0.05.

### 4.13. Cell-Type Prioritization by Integrating Multi-Modal Evidence

To rank cell types most likely implicated in NAFLD and hypothyroidism, we integrated evidence from three pipelines—single-cell atlas annotation, ECLIPSER, and CELLECT—assigning one point to each source. Scores were summed, with higher totals indicating a greater likelihood of disease relevance [63].

### 4.14. High-Dimensional Weighted Gene Co-Expression Network Analysis in Prioritized Cell Types

We applied high-dimensional Weighted Gene Co-expression Network Analysis (hdWGCNA) to cell types prioritized by the integrated framework. Annotated Seurat objects were subset to the target cell types. Genes expressed in ≥5% of cells were retained. Metacells (k = 25) were constructed by cell type and sample to alleviate sparsity while preserving biological variance; metacell expression was normalized and standardized. After PCA and Harmony batch correction, we extracted expression matrices, evaluated soft-threshold powers, and constructed signed topological overlap networks. Co-expression modules were identified via dynamic tree cutting, module eigengenes were computed, module–cell-type associations and connectivity were quantified, and modules were renamed to aid interpretation.

### 4.15. Colocalization with eCAVIAR

To nominate high-confidence genes and regulatory mechanisms (eQTL/sQTL) mediating shared GWAS signals for NAFLD and hypothyroidism, we employed eCAVIAR, a Bayesian colocalization method that models LD and allelic heterogeneity and includes fine-mapping functionality [64]. Inputs were Z-scores (effect size divided by standard error) from GWAS and GTEx e/sQTL studies. For each index GWAS variant, the LD window encompassed variants with r^2^ > 0.1 (1000 Genomes Phase 3 reference), extended ±50 kb. We allowed up to two independent causal variants per locus. Colocalizations with CLPP > 0.01 were considered significant.

### 4.16. Colocalization with fastENLOC

We further performed Bayesian colocalization using fastENLOC (version 3.2) [65], which fine-maps GWAS and e/sQTL loci via DAP-G to estimate per-variant posterior causal probabilities and evaluates shared causality without pre-specifying the number of causal variants per locus. Inputs were Z-scores from GWAS and GTEx e/sQTL summary statistics. LD windows were defined as for eCAVIAR (r^2^ > 0.1; ±50 kb; 1000 Genomes Phase 3). Colocalization was summarized by regional colocalization probability (RCP), with RCP > 0.1 considered significant.

### 4.17. Linking Open Chromatin to Gene Expression

To interrogate links between chromatin accessibility and gene expression in immune cells from normal tissues and assess potential overlap with NAFLD/hypothyroidism cell annotations, we used Open4Gene, a Hurdle model-based framework tailored to zero-inflated single-cell data. Using Seurat and Signac, scRNA-seq and scATAC-seq datasets were normalized, reduced, and clustered. From Seurat objects, we extracted RNA expression matrices, ATAC peak matrices, and cell metadata (including annotations and technical covariates). ATAC peaks were associated with gene promoter regions, and peak–gene pairs were defined within 100 kb. A two-part Hurdle model tested each peak–gene pair: a logistic component modeled the relationship between accessibility and zero expression probability, and a truncated negative binomial component modeled the association between accessibility and nonzero expression levels [66].

### 4.18. Mapping and Annotation of Genomic Risk Loci

Genomic risk loci were identified and annotated using FUMA (https://fuma.ctglab.nl/snp2gene) (accessed on 25 July 2025). We uploaded NAFLD and hypothyroidism GWAS files with SNP identifiers and reference LD information. FUMA performed QC to remove missing and low-quality variants, and genome-wide significant loci were defined at *p* < 5 × 10^−8^ (adjustable as needed).

### 4.19. Conditional Analysis of Genomic Risk Signals

To assess secondary signals within colocalized GWAS loci, we conducted conditional analyses using GCTA-COJO (version 1.94.1) [67], conditioning on the lead variant per locus. Variants with MAF < 1 × 10^−4^ were excluded to retain low-frequency leads. Allele frequencies and LD were derived from the 1000 Genomes Project.

### 4.20. Cell-Level Expression Annotation and Exon-Level Expression Profiling of Prioritized Genes

We visualized cell composition across samples with and grouped UMAPs and annotated cell-level expression of prioritized genes (from hdWGCNA, eCAVIAR, fastENLOC, and Open4Gene) using UMAP overlays. All outputs were exported as high-resolution figures and structured tables. Exon-level expression from GTEx was used to annotate transcript isoforms, structural features, and tissue-specific distributions for prioritized genes.

## 5. Conclusions

Overall, multi-omics integration anchors the shared genetic risk of NAFLD and hypothyroidism in hepatocytes, implicating core pathways of energy and lipid metabolism. Guided by spatial omics, we advance a spatiotemporal two-hit model. We also prioritize MAGI3, RRNAD1, and PRCC, together with the sentinel locus rs926103, as actionable targets and testable leads for functional validation and precision intervention. Collectively, these findings furnish a conceptual and methodological roadmap for early screening and therapy in NAFLD populations comorbid with hypothyroidism and provide an experimentally tractable, dynamic framework for interrogating the full pathophysiological continuum linking the two disorders.

## Figures and Tables

**Table 1:** Cell-type enrichment of e/sQTL-mapped genes for NAFLD and hypothyroidism GWAS locus sets in liver and thyroid using ECLIPSER.

Trait	Tissue	Cell Type	Fold-Enrichment	Fold-Enrichment (95% CI)	*p*
NAFLD	Liver	Hepatocytes	0.84	0.40, 0.99	0.999
NAFLD	Liver	Monocyte	7.61	1.33, 16.27	0.014
NAFLD	Liver	T cells	0.84	0.40, 0.99	0.999
Hypothyroidism	Thyroid	Fibroblasts	2.58	0.81, 5.65	0.049
Hypothyroidism	Thyroid	Macrophage	0.97	0.86, 1.00	0.991
Hypothyroidism	Thyroid	Tissue stem cells	2.27	0.71, 4.94	0.073

NAFLD, nonalcoholic fatty liver disease; CI, confidence interval.

**Table 2:** Cell-type prioritization in NAFLD and hypothyroidism.

Cell Type	Tissue	Cell Atlas	ECLIPSER	CELLECT	Total
Hepatocytes	Liver	1	0	2	3
Monocyte	Liver	1	1	0	2
Natural Killer Cell	Liver	0	0	2	2
T cells	Liver	1	0	0	1
B cell	Liver	0	0	1	1
Fibroblasts	Thyroid	1	1	0	2
Macrophage	Thyroid	1	0	0	1
B cell	Thyroid	1	0	0	1
Tissue stem cells	Thyroid	1	0	0	1
Dendritic Cell	Thyroid	1	0	0	1

NAFLD, nonalcoholic fatty liver disease.

**Table 3:** Evaluation table of candidate gene for NAFLD and hypothyroidism.

Method	Level of Evidence: High	Level of Evidence: Medium	Level of Evidence: Low
hdWGCNA	YES/NO	YES/NO	YES/NO
eCAVIAR	CLPP ≥ 0.8	CLPP ≥ 0.7	CLPP ≥ 0.01
fastenloc	RCP ≥ 0.9	RCP ≥ 0.7	RCP ≥ 0.1
Open4Gene	YES	YES/NO	YES/NO

NAFLD, nonalcoholic fatty liver disease; CLPP, colocalization posterior probability; RCP, regional colocalization probability.

## Data Availability

The datasets analyzed during the current study are available in the finngen database repository, https://www.finngen.fi/en (accessed on 22 June 2025) (pheno: Nonalcoholic fatty liver disease). The datasets analyzed in the current study are available in the GWAS Catalog repository, https://www.ebi.ac.uk/gwas/home (accessed on 22 June 2025) (ID: GCST90054782). The datasets analyzed in the current study are available in the IEU OpenGWAS repository, https://opengwas.io/ (accessed on 22 June 2025) (ID: ukb-e-244_CSA). The datasets analyzed in the current study are available in the GWAS Catalog repository, https://www.ebi.ac.uk/gwas/home (accessed on 22 June 2025) (ID: GCST90435692). The datasets analyzed in the current study are available in the GEO repository, https://www.ncbi.nlm.nih.gov/geo/ (accessed on 15 August 2025) (IDs: GSE202379 and GSE248205). The datasets analyzed during the current study are available in the 10× Genomics repository, https://www.10xgenomics.com/datasets/pbmc-from-a-healthy-donor-granulocytes-removed-through-cell-sorting-10-k-1-standard-1-0-0 (accessed on 25 August 2025) (single-cell multi-omics data from healthy human PBMCs). The datasets analyzed in the current study are available in the GTEx repository, https://gtexportal.org/home/datasets (accessed on 30 August 2025) (eQTL and sQTL summary statistics). The datasets analyzed in the current study are available in the MSigDB repository, http://www.gsea-msigdb.org/gsea/msigdb/collections.jsp (accessed on 16 July 2025) (Gene Ontology, Reactome, and KEGG gene sets). The datasets analyzed in the current study are available in the Mouse Genome Informatics (MGI) repository, http://www.informatics.jax.org/ (accessed on 16 July 2025) (Mouse Phenotype Ontology gene sets).

## Supplementary Materials

The following supporting information can be downloaded at https://www.mdpi.com/article/10.3390/ijms27041850/s1.

## Abbreviations

HPT	hypothalamic–pituitary–thyroid
ATAC-seq	Assay for Transposase Accessible Chromatin with high-throughput sequencing
BP	Biological Processes
CC	Cellular Components
ChREBP	Carbohydrate-responsive element-binding protein
CLPP	colocalization posterior probability
D2	type 2 iodothyronine deiodinase
eQTLs	expression quantitative trait loci
FAT (CD36)	fatty acid translocase (cluster of differentiation 36)
FATPs	fatty acid transport proteins
FFAs	free fatty acids
GO	Gene Ontology
gsMap	genetically informed spatial mapping
GWAS	genome-wide association studies
hdWGCNA	high-dimensional Weighted Gene Co-expression Network Analysis
KEGG	Kyoto Encyclopedia of Genes and Genomes
kME	Module Eigengene-based Connectivity
LD	linkage disequilibrium
LDL	low-density lipoprotein
L-FABPs	liver-type fatty acid-binding proteins
LXR	Liver X Receptor
MAGMA	Multi-marker Analysis of GenoMic Annotation
MASH	metabolic dysfunction-associated steatohepatitis
MASLD	metabolic dysfunction-associated steatotic liver disease
MF	Molecular Function
MGI	Mouse Genome Informatics
MSigDB	Molecular Signatures Database
NAFLD	nonalcoholic fatty liver disease
NASH	nonalcoholic steatohepatitis
PBMC	Peripheral Blood Mononuclear Cell
PCA	Principal Component Analysis
QC	quality control
RCP	regional colocalization probability
scRNA-seq	single-cell RNA sequencing
sc-ST	single-cell spatial transcriptomics
S-LDSC	Stratified LD Score Regression
SNPs	single-nucleotide polymorphisms
sQTLs	splicing quantitative trait loci
T2	3,5-diiodothyronine
T3	triiodothyronine
T4	thyroxine
TH/TR	thyroid hormone/receptor
THR-β	thyroid hormone receptor-β
WAT	white adipose tissue
